# Cytological DNA methylation for cervical cancer screening: a validation set

**DOI:** 10.3389/fonc.2023.1181982

**Published:** 2023-08-21

**Authors:** Linghua Kong, Linhai Wang, Ziyun Wang, Xiaoping Xiao, Yan You, Huanwen Wu, Ming Wu, Pei Liu, Lei Li

**Affiliations:** ^1^ Department of Obstetrics and Gynecology, National Clinical Research Center for Obstetric & Gynecologic Diseases, Peking Union Medical College Hospital, Beijing, China; ^2^ Department of Technology, Beijing OriginPoly Biotechnology CO., Ltd., Beijing, China; ^3^ Department of Pathology, Peking Union Medical College Hospital, Beijing, China

**Keywords:** cervical cancer, cervical intraepithelial neoplasia, DNA methylation, high-risk human papillomavirus, validation set, endometrial carcinoma

## Abstract

**Background:**

In a previous training set with a case-controlled design, cutoff values for host *EPB41L3* and *JAM3* gene methylation were obtained for the detection of cervical intraepithelial neoplasia (CIN) 2 or more severe lesions (CIN2+). This validation trial was conducted to evaluate the role of DNA methylation in screening for CIN2+ by cervical cytology among unselected participants.

**Methods:**

From June 1, 2019, to September 1, 2019, in our study center, we collected liquid-based samples from cervical swabs for methylation assays and hrHPV testing in eligible patients. The primary endpoint was the diagnostic accuracy of DNA methylation and hrHPV genotyping for CIN2+ according to confirmed histology results.

**Results:**

Among 307 participants, compared with hrHPV testing, the methylation assay for CIN2+ had lower sensitivity (68.7% versus 86.1%, *p*=0.002) but higher specificity (96.7% versus 0.696, *p<*0.001). The methylation assay also had favorable sensitivity and specificity in patients with negative hrHPV testing (56.3% and 96.9%) and in patients with cervical adenocarcinoma (73.7% and 92.7%). DNA methylation had higher specificity than the hrHPV assay (100.0% versus 44.4%, *p<*0.001) for identifying residual CIN2+ in patients without residual lesions. Positive cervical DNA methylation was associated with a diagnostic probability of endometrial carcinoma (odds ratio 15.5 [95% confidence interval 4.1-58.6]) but not of ovarian epithelial carcinoma (1.4 [0.3-6.5]).

**Conclusions:**

The host *EPB41L3* and *JAM3* gene methylation assay in cervical cytology had favorable diagnostic accuracy for CIN2+ and was highly specific for residual CIN2+ lesions The methylation assay is a promising triage tool in hrHPV+ women, or even an independent tool for cervical cancer screening. The methylation status in cervical cytology could also serve as a prognostic biomarker. Its role in detecting endometrial carcinomas is worthy of further exploration.

## Introduction

Uterine cervical cancer is one of the most common causes of cancer-related deaths among women globally (1) and in China (2), and Chinese patients account for 28% of the total number of new cases of cervical cancer worldwide (3). Robust and standard screening programs would significantly decrease the incidence of cervical cancer (4). Currently, cervical cytology and/or high-risk human papillomavirus (hrHPV) testing are the main screening methods (5, 6). However, both cytology and hrHPV testing have limitations in terms of their diagnostic accuracy (7, 8). A cost-benefit strategy with high accuracy and feasibility is essential for decision making in cervical cancer screening (9) and is also urgently needed in developing countries, such as China.

DNA methylation is an epigenetic mechanism that results in heritable silencing of genes without changes to their coding sequences (10, 11). More than 100 human (host) genes have been reported to be possible methylation biomarkers of cervical cancer (12), and several of them had been validated for the correlation with cervical cancer development (13). Changes in histone modification at HPV integration events were correlated with the upregulation of nearby genes and endogenous retroviruses (14). Genotyping and methylation markers are objective and can be used with self-obtained samples (9), even with urine samples (15), offering great advantages in low- and middle-income settings. Numerous studies have shown that methylation has favorable screening sensitivity for cervical intraepithelial neoplasia (CIN) 2 or more severe lesions (CIN2+, or high-grade intraepithelial lesions [HSIL]) as a triage method for women with positive hrHPV status. Multiple panels have been utilized as classifiers consisting of dozens of candidate host genes, viral genes or both, as well as various combinations thereof (16). Methylation testing has been recommended as one of the future approaches for cervical cancer screening by the World Health Organization (17) and other guidelines (18). However, most studies have addressed only the triage role of methylation assays in cervical cancer screening programs, rather than their independent diagnostic capabilities. The impact of prior cervical procedures and uterine or ovarian diseases on cytology methylation assays has also been scarcely investigated.

The JAM (Junctional Adhesion Molecule) family, a part of the immunoglobulin superfamily, has a direct impact on the tight junction function of epithelial and endothelial cells (19). JAM3, in particular, has been extensively studied as a regulator of adhesion and transmigration (20). Recent research has shed light on the crucial role of JAM3 in the regulation of tumor growth during tumor progression (21). Erythrocyte Membrane Protein Band 4.1 Like 3 (EPB41L3), also known as Protein 4.1B/DAL-1, is a membrane skeletal protein with involvement in various cytoskeleton-associated processes. Its functions encompass cell motility, adhesion, growth, and differentiation (22). EPB41L3 plays a crucial role in inhibiting cell growth by inducing apoptosis (programmed cell death) and cell cycle arrest (23). Through these mechanisms, EPB41L3 exerts regulatory control over cellular processes, contributing to the overall balance and homeostasis of cell behavior (24). Functionally, EPB41L3 inhibits cell growth by inducing apoptosis and cell cycle arrest (25). In our previous exploratory study (26), the cutoff values of *EPB41L3* and *JAM3* methylation assays were obtained for detecting CIN2+ in case-controlled studies, and their favorable performance suggested that DNA methylation could be the preferred screening method regardless of hrHPV status. In this validation set, *EPB41L3* and *JAM3* gene methylation assays and hrHPV genotyping were evaluated in cervical liquid-based samples before conization or hysterectomy from unselected patients with various gynecological diseases in a gynecologic oncology unit. The primary objective was to determine the diagnostic accuracy of various screening strategies. The secondary objective was to determine the effects of previous biopsy or conization on methylation results, as this may have a significant impact on the residual cervical lesions caused by uterine or ovarian diseases.

## Methods

### Ethical approval

The institutional review board of our study center approved the study (No. JS-1954). All of the patients provided their consent before enrollment. The registration number is NCT03960879 (*clinicaltrials.gov*, registered on May 23, 2019). All of the procedures in the study involving human participants were in accordance with the ethical standards of the institutional and national research committees and with the 1964 *Declaration of Helsinki* and its later amendments or comparable ethical standards.

### Study design

The study was performed in a prospective cohort of patients with indications for conization, the loop electrosurgical excision procedure (LEEP) or hysterectomy for various gynecologic diseases. In such situations, cervical histology could be evaluated sufficiently. Before surgery, liquid-based samples were collected from cervical swabs and analyzed for both DNA methylation and hrHPV genotyping. The accuracies of the DNA methylation assay, hrHPV genotyping and their combination were compared for various surgically confirmed cervical pathological types. The primary endpoints were the diagnostic accuracies of DNA methylation and hrHPV testing for CIN2+ in liquid-based cytology specimens. The secondary endpoints were the diagnostic accuracies of DNA methylation and hrHPV testing for CIN2+ with or without residual lesions and for specific pathological types, including cervical adenocarcinomas (ADCs) and endometrial and ovarian tumors.

### Patient enrollment

This study enrolled eligible patients in one gynecologic oncology unit of the study center from June 1, 2019, to September 1, 2019. Data regarding the patients’ demographic characteristics and medical histories were obtained from the medical records and supplemented by interviews with the patients. The inclusion criteria were as follows: aged 18 years or older; consent for conization, LEEP or hysterectomy, with relevant comprehensive pathological results obtained; negative HIV results, no history of organ transplantation or usage of immunosuppressive therapy; and willingness to participate in the study. Cases not meeting all of the criteria were excluded. All of the cervical histological materials were re-evaluated by two pathologists (YY and HW). For patients with CIN2/3 or cervical cancer as the primary diagnosis, pathological results before and after cervical biopsy, conization or LEEP were checked meticulously to confirm whether there were residual lesions because biopsy or excision before the last surgery would likely have eliminated the primary lesions.

### Collection and assays of study materials

One day before surgery, a liquid-based sample was collected from cervical swabs and stored in PreservCyt Solution (Thinprep Pap Test; Hologic, Marlborough, MA, USA) at room temperature by the medical staff. The assays for DNA methylation and hrHPV have been described previously (26). Methylation of the *EPB41L3* and *JAM3* genes was evaluated using TaqMan-based technologies with the Methylated Human *EPB43* and *JAM3* Gene Detection kit (real-time fluorescent polymerase chain reaction [PCR], developed by Beijing SinoMDgene Technology Co., Ltd., China) and an ABI 7300 Real Time Fluorescence Quantitative PCR system (Life Tech, USA). The methylation level of each gene was determined by the ΔCt value (the target gene Ct value subtracts the reference gene Ct value). Positive status was defined as ΔCt values less than 7.945 and 9.250 for *EPB41L3* and *JAM3*, respectively, according to the results from previous training sets (26). Total methylation status was defined as positive *EPB41L3* and/or *JAM3* methylation.

hrHPV genotyping was performed with TaqMan-based technology using an ABI 7500 Real Time Fluorescence Quantitative PCR system (Life Tech, USA) or a Stratagene Mx3000p Fluorescence Quantitative PCR system (Stratagene, USA) with an HPV nucleic acid genotyping diagnostic kit (Real time Fluorescent PCR developed by Beijing SinoMDgene Technology Co., Ltd., China). The diagnostic kit detects a pooled result for hrHPV types, including HPV 16, 18, 31, 33, 45, 52, 6, 11, 35, 51, 39, 59, 68, 56, 58, and 66, with type-specific probes.

### Statistics

Nonnormally distributed variables and categorical data were compared between different screening groups using nonparametric tests. The specificity, sensitivity, negative predictive value (NPV), and positive predictive value (PPV) were also calculated for various screening groups. The odds ratios (ORs) and 95% confidence intervals (95% CIs) of the positive ratios of different screening methods for various histological types were calculated with logistic regression models. Unless otherwise stated, all of the analyses were performed with a two-sided significance level of 0.05 and were conducted with the use of Statistical Product and Service Solutions Statistics software, version 20.0 (IBM Corporation, Armonk, NY, USA).

## Results

### Patient characteristics

**Figure 1:**
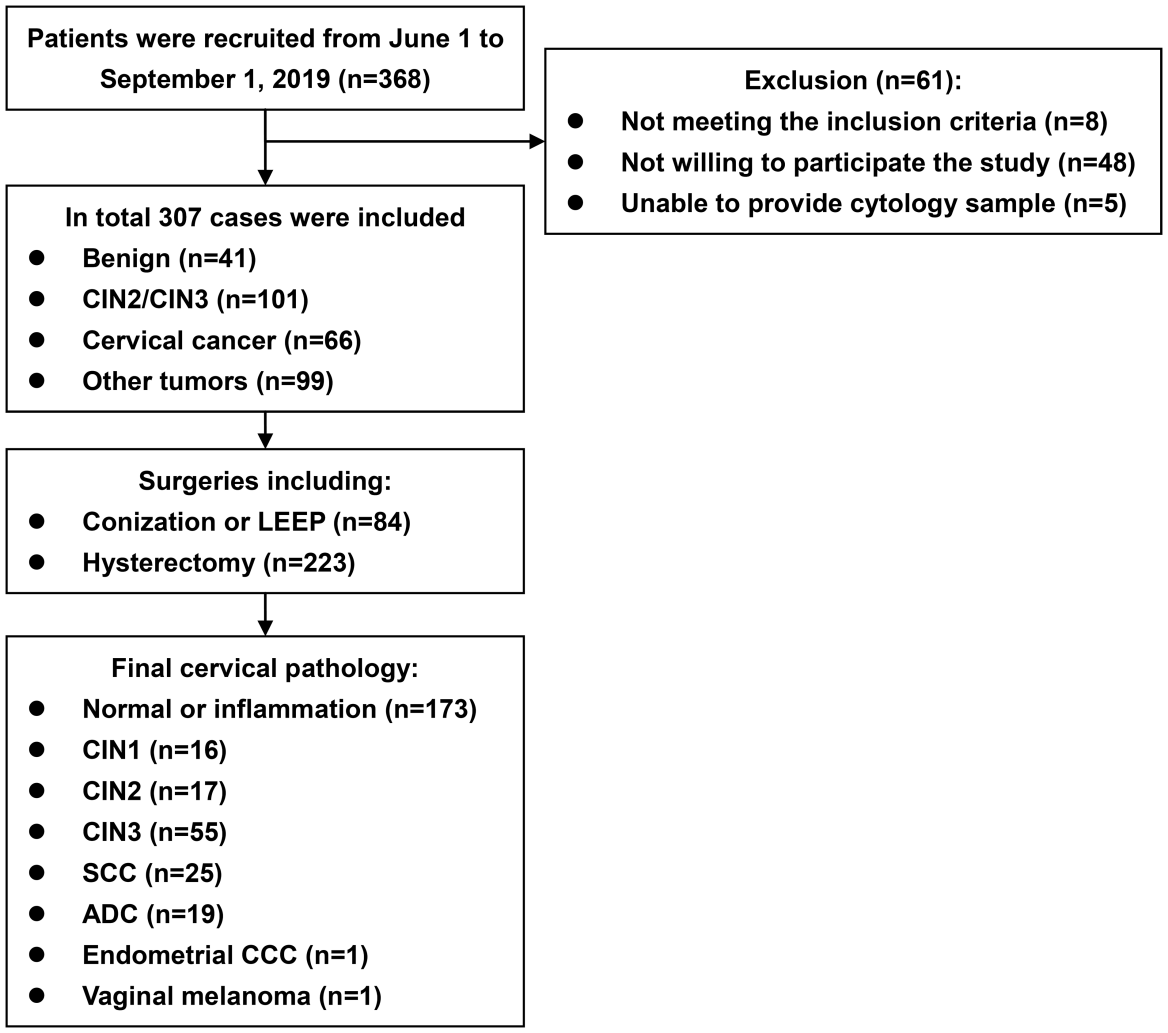
Flow diagram of the study. ADC, adenocarcinoma. CCC, clear cell carcinoma. CIN, cervical intraepithelial neoplasm. LEEP, loop electrosurgical excision procedure. SCC, squamous cell carcinoma.

A flow diagram of the study is provided in the **Figure 1**. During the study period, 368 eligible patients were recruited, and 307 were included. The median age was 46 years (range 22 to 77). Regarding primary diagnoses, there were 41 cases of benign ovarian or uterine disease, 101 of CIN2/3, 66 of cervical cancer, and 99 of ovarian or uterine malignancies, precancerous lesions, or borderline tumors. The surgeries consisted of 84 cases of conization or LEEP and 223 hysterectomies. The final cervical pathology results are listed in **Table 1** and the **Figure 1**. The median interval from biopsy to conization/LEEP or from conization/LEEP to hysterectomy/radical hysterectomy was 40 days (range 10 to 51). DNA methylation and hrHPV assays were successfully performed for all 307 patients. There were 68 (22.1%), 103 (33.6%), 109 (35.5%), 131 (42.7%), and 72 (23.5%) cases with positive *EPB41L3*, *JAM3*, *EPB41L3* or *JAM3*, hrHPV testing and methylation assay plus hrHPV testing, respectively. Patients with positive and negative methylation had similar average ages (45.9 ± 10.0 versus 47.9 ± 12.1 years old, *p*=0.143), but patients with positive hrHPV testing were significantly younger than patients with negative hrHPV testing (42.8 ± 10.5 versus 50.4 ± 11.1 years old, *p<*0.001).

**Table 1:** Cervical procedures and pathology before and after sampling for DNA methylation.

Primary diagnosis	Cervical procedures before DNA methylation (n)			Surgeries after DNA methylation (n)		Actual cervical pathology after DNA methylation
	None	Biopsy	Conization or LEEP	Conization or LEEP	Hysterectomy	
Benign (n=41)	41	0	0	0	41	Inflammation (n=40) CIN1 (n=1)
CIN2/3 (n=101)	0	84	17	84	17	Inflammation (n=16) CIN1 (n=13) CIN2 (n=17) CIN3 (n=55)
Cervical carcinomas (n=66)	0	37	29	0	65	Inflammation (n=21) CIN1 (n=1) SCC (n=25) ADC, villoglandular type (n=3) ADC, in situ (n=6) ADC, endocervical type (n=9) ADC, mucinous type (n=1)
Others* (n=99)	99	0	0	0	99	Inflammation (n=96) CIN1 (n=1) Endometrial clear cell carcinoma (n=1) Vaginal melanoma (n=1)

*Others includes gynecological malignancies, precancerous lesions, or borderline tumors rather than cervical lesions.

Three cases were excluded from the table, including one cervical mucinous adenocarcinoma, one uterine clear cell carcinoma, and one vaginal melanoma. ADC, adenocarcinoma. CIN, cervical intraepithelial lesion. LEEP, loop electrosurgical excision procedure. SCC, squamous cell carcinoma.

### Diagnostic accuracies for cervical lesions

The diagnostic accuracies of the various screening methods are listed in **Table 2**. For the diagnosis of CIN2+, the sensitivities, specificities, PPVs and NPVs of the methylation assay in the previous training set (26) and in the current trial were similar, namely, 72.1% versus 68.7% (*p*=0.525), 91.5% versus 96.7% (*p*=0.120), 0.679 versus 0.712 (*p*=0.232), and 0.930 versus 0.963 (*p*=0.552), respectively. As shown in **Table 2**, for the diagnosis of CIN2+, assays for methylation, hrHPV, HPV 16/18 and their combination all had favorable results, with ORs of 69.105 (95% CI 19.294-219.611), 14.143 (7.094-28.197), 7.953 (4.118-15.362) and 141.556 (19.043-1052.225), respectively.

**Table 2:** The diagnostic accuracies of DNA methylation, hrHPV and their combinations for the final cervical pathology.

Cervical pathology	Positive DNA methylation			Positive hrHPV			PositiveHPV16 or HPV18			Positive DNA methylation and hrHPV		
	n (%)	OR (95% CI)	*p*	n (%)	OR (95% CI)	*p*	n (%)	OR (95% CI)	*p*	n (%)	OR (95% CI)	*p*
Normal in benign diseases* (n=77)	3 (3.9)	Reference	–	18 (23.4)	Reference	–	11 (14.3)	Reference	–	1 (1.3)	Reference	–
Normal in others† (n=96)	25 (26.0)	8.685 (2.511-30.405)	**0.001**	3 (3.1)	0.106 (0.030-0.375)	**<0.001**	0 (0.0)	0.000 (0.000-N/A)	0.996	0 (0.0)	0.000 (0.000-N/A)	0.997
CIN1 (n=15)	0 (0.0)	0.000 (0.000-N/A)	0.999	10 (66.7)	6.556 (1.982-21.683)	**0.002**	5 (33.3)	3.000 (0.860-10.460)	0.085	0 (0.0)	0.000 (0.000-N/A)	0.999
CIN2 (n=17)	3 (17.6)	5.286 (0.966-28.911)	0.055	13 (76.5)	10.653 (3.087-36.764)	**<0.001**	6 (35.3)	3.273 (1.004-10.671)	**0.049**	2 (11.8)	10.133 (0.863-119.205)	0.065
CIN3 (n=55)	38 (69.1)	55.137 (15.205-199.941)	**<0.001**	50 (90.9)	32.778 (11.356-94.611)	**<0.001**	33 (60.0)	9.000 (3.902-20.759)	**<0.001**	35 (63.6)	133.000 (17.158-1030.948)	**<0.001**
SCC (n=25)	24 (96.0)	592.00 (58.790-5961.242)	**<0.001**	22 (88.0)	24.037 (6.443-89.682)	**<0.001**	21 (84.0)	31.500 (9.068-109.428)	**<0.001**	22 (88.0)	557.333 (55.190-5628.193)	**<0.001**
ADC (n=18)	14 (77.8)	86.333 (17.392-428.568)	**<0.001**	14 (77.8)	11.472 (3.353-39.255)	**<0.001**	12 (66.7)	12.000 (3.726-38.646)	**<0.001**	11 (61.1)	119.429 (13.385-1065.636)	**<0.001**
Normal* or CIN1 (n=92)	3 (3.3)	Reference	–	28 (30.4)	Reference	–	16 (17.4)	Reference	–	1 (1.1)	Reference	–
CIN2+ (n=115)	79 (68.7)	65.105 (19.294-219.611)	**<0.001**	99 (86.1)	14.143 (7.094-28.197)	**<0.001**	72 (62.6)	7.953 (4.118-15.362)	**<0.001**	70 (60.9)	141.556 (19.043-1052.225)	**<0.001**
Sensitivity (%)	68.7			86.1			62.6			60.9		
Specificity (%)	96.7			69.6			82.6			98.9		
PPV	0.963			0.780			0.818			0.986		
NPV	0.712			0.800			0.639			0.669		

*These cases excluded gynecological malignancies, precancerous lesions, or borderline tumors rather than cervical lesions.

†These cases only included gynecological malignancies, precancerous lesions, or borderline tumors rather than cervical lesions.

ADC, adenocarcinoma. CIN2+, cervical intraepithelial neoplasia (CIN) 2 or more severe lesion. hrHPV, high-risk human papillomavirus. N/A, not available. NPV, negative predictive value. OR, odds ratio. 95% CI, 95% confidence interval. PPV, positive predictive value. SCC, squamous cell carcinoma.The bold values mean values with significant statistical meaning.

As shown in **Table 2**, the sensitivity value of the methylation assay was lower than that of hrHPV testing (68.7% versus 86.1%, *p*=0.002) but similar to that of HPV 16/18 testing (68.7% versus 62.6%, *p*=0.331). The specificity value of the methylation assay was significantly higher than that of hrHPV testing (96.7% versus 0.696, *p*<0.001) or that of HPV 16/18 testing (96.7% versus 82.6%, *p*=0.002). The PPV of the methylation assay was significantly higher than that of hrHPV testing (0.963 versus 0.780, *p<*0.001) or that of HPV 16/18 testing (0.963 versus 0.818, *p*=0.003). The NPV of the methylation assay was similar to that of hrHPV testing (0.712 and 0.669 versus 0.800, *p*=0.158) and that of HPV 16/18 testing (0.712 versus 0.639, *p*=0.221).

Even in patients with negative hrHPV results, DNA methylation still had a significant discrepancy for CIN2+ (OR 39.857, 95% CI 7.137-222.577). In this population, the sensitivity, specificity, PPV and NPV of the DNA methylation assay were 56.3%, 96.9%, 0.818 and 0.899, respectively.

As shown in **Table 3**, in patients without residual SCC and CIN2/3 lesions after biopsy or excision, their methylation status was similar to those with benign uterine and ovarian lesions (*p*=0.999 and 0.998), but the positive hrHPV ratios were all significantly higher (*p*=0.002 and <0.001). For identifying CIN2+ residual lesions in CIN2+ patients without residual lesions, when the DNA methylation assay and hrHPV testing were compared, the sensitivity, specificity, PPV and NPV were 67.0% versus 88.6% (*p*=0.001), 100.0% versus 44.4% (*p<*0.001), 1.000 versus 0.773 (*p<*0.001), and 0.584 versus 0.625 (*p*=0.694), respectively.

**Table 3:** The diagnostic accuracies of DNA methylation, hrHPV and their combination for cervical SCC and CIN2/3 with or without residual lesions.

Cervical pathology	Positive DNA methylation			Positive hrHPV			Positive DNA methylation and hrHPV		
	n (%)	OR (95% CI)	*p*	n (%)	OR (95% CI)	*p*	n (%)	OR (95% CI)	*p*
Normal* (n=41)	3 (7.3)	Reference	–	2 (4.9)	Reference	–	1 (2.4)	Reference	–
SCC without residual lesions (n=16)	0 (0.0)	0.000 (0.000-N/A)	0.999	7 (43.8)	15.167 (2.687-85.598)	**0.002**	0 (0.0)	0.000 (0.000-N/A)	0.999
SCC with residual lesions (n=25)	24 (96.1)	304.000 (29.871-3093.870)	**<0.001**	22 (88.0)	143.000(22.173-922.234)	**<0.001**	22 (88.0)	293.333 (28.764-2991.426)	**<0.001**
CIN2/CIN3 without residual lesions (n=29)	0 (0.0)	0.000 (0.000-N/A)	0.998	18 (62.1)	31.909 (6.399-159.128)	**<0.001**	0 (0.0)	0.000 (0.000-N/A)	0.998
CIN2/CIN3 with residual lesions (n=72)	41 (56.9)	16.753 (4.730-59.332)	**<0.001**	63 (87.5)	136.500 (28.021-664.933)	**<0.001**	37 (51.4)	42.286 (5.513-324.365)	**<0.001**

*These cases only included uterine or ovarian benign diseases.

CIN, cervical intraepithelial neoplasia. hrHPV, high-risk human papillomavirus. N/A, not available. OR, odds ratio. 95% CI, 95% confidence interval. SCC, squamous cell carcinoma.The bold values mean values with significant statistical meaning.

For the diagnosis of cervical ADC, methylation assays, hrHPV testing and their combination all had favorable results (**Table 4**). According to various subtypes of ADC, compared with uterine or ovarian benign diseases, methylation assays (OR 44.333, 95% CI 6.230-315.499), hrHPV testing (156.000, 12.575-1935.197) and their combination (80.000, 7.111-900.008) had the highest ORs in endocervical ADC, which was the most common pathology in this cohort.

**Table 4:** The diagnostic accuracies of DNA methylation, hrHPV and their combination for cervical adenocarcinoma.

Cervical pathology	Positive DNA methylation			Positive hrHPV			Positive DNA methylation and hrHPV		
	n (%)	OR (95% CI)	*p*	n (%)	OR (95% CI)	*p*	n (%)	OR (95% CI)	*p*
Normal* (n=41)	3 (7.3)	Reference	–	2 (4.9)	Reference	–	1 (2.4)	Reference	–
ADC, villoglandular type (n=3)	3 (100.0)	N/A (0.000-N/A)	0.999	2 (66.7)	39.000 (2.397-634.654)	**0.010**	2 (66.7)	80.000 (3.552-1801.650)	**0.006**
ADC, in situ (n=6)	4 (66.7)	25.333 (3.214-199.68)	**0.002**	4 (66.7)	39.000 (4.263-356.819)	**0.001**	3 (50.0)	40.000 (3.126-511.879)	**0.005**
ADC, endocervical type (n=9)	7 (77.8)	44.333 (6.230-315.499)	**<0.001**	8 (88.9)	156.000 (12.575-1935.197)	**<0.001**	6 (66.7)	80.000 (7.111-900.008)	**<0.001**
ADC, mucinous type (n=1)	0 (0.0)	0.000 (0.000-N/A)	1.000	0 (0.0)	0.000 (0.000-N/A)	1.000	0 (0.0)	0.000 (0.000-N/A)	1.000
Normal* (n=41)	3 (7.3)	Reference	–	2 (4.9)	Reference	–	1 (2.4)	Reference	–
ADC (n=19)	14 (73.7)	35.467 (7.475-168.274)	**<0.001**	14 (73.7)	54.600 (9.490-314.148)	**<0.001**	11 (57.9)	55.000 (6.197-488.166)	**<0.001**
Sensitivity (%)	73.7			73.7			57.9		
Specificity (%)	92.7			95.1			97.6		
PPV	0.824			0.875			0.917		
NPV	0.884			0.886			0.833		

*These cases only included uterine or ovarian benign diseases.

ADC, adenocarcinoma. hrHPV, high-risk human papillomavirus. N/A, not available. NPV, negative predictive value. OR, odds ratio. 95% CI, 95% confidence interval. PPV, positive predictive value. SCC, squamous cell carcinoma.The bold values mean values with significant statistical meaning.

### Diagnostic accuracies for ovarian or uterine diseases

The diagnostic accuracies for ovarian or endometrial tumors are listed in **Table 5**. Positive methylation was found in 12.2% (5/41) of ovarian epithelial cancers and 55.0% (22/40) of endometrial carcinomas, corresponding to ORs of 1.435 (95% CI 0.317-6.495) and 15.481 (4.093-58.552) for detecting malignancies, respectively. hrHPV testing could not differentiate endometrial carcinomas or ovarian cancers from their benign counterparts.

**Table 5:** The diagnostic accuracies of DNA methylation, hrHPV and their combination for endometrial and ovarian lesions.

Cervical pathology	Positive DNA methylation			Positive hrHPV			Positive DNA methylation and hrHPV		
	n (%)	OR (95% CI)	*p*	n (%)	OR (95% CI)	*p*	n (%)	OR (95% CI)	*p*
Normal* (n=34)	3 (8.8)	Reference	–	2 (5.9)	Reference	–	1 (2.9)	Reference	–
Ovarian benign diseases (n=7)	0 (0.0)	0.000 (0.000-N/A)	0.999	0 (0.0)	0.000 (0.000-N/A)	0.999	0 (0.0)	0.000 (0.000-N/A)	0.999
Ovarian borderline diseases (n=4)	0 (0.0)	0.000 (0.000-N/A)	0.999	0 (0.0)	0.000 (0.000-N/A)	0.999	0 (0.0)	0.000 (0.000-N/A)	0.999
Ovarian epithelial carcinomas (n=41)	5 (12.2)	1.435 (0.317-6.495)	0.639	1 (2.4)	0.400 (0.035-4.612)	0.463	0 (0.0)	0.000 (0.000-N/A)	0.998
Normal† (n=41)	3 (7.3)	Reference	–	2 (4.9)	Reference	–	1 (2.4)	Reference	–
Endometrial intraepithelial neoplasms (n=7)	0 (0.0)	0.000 (0.000-N/A)	0.999	1 (14.3)	3.250 (0.254-41.610)	0.365	0 (0.0)	0.000 (0.000-N/A)	0.999
Endometrial carcinomas (n=40)	22 (55.0)	15.481 (4.093-58.552)	**<0.001**	1 (2.5)	0.500 (0.044-5.743)	0.578	1 (2.5)	1.026 (0.062-16.979)	0.986

*These cases only included benign diseases except for ovarian diseases.

†These cases only included benign diseases.

hrHPV, high-risk human papillomavirus. N/A, not available. OR, odds ratio. 95% CI, 95% confidence interval.The bold values mean values with significant statistical meaning.

## Discussion

A number of studies have explored the role of a panel that includes *EPB41L3* (27–37), *JAM3* (38), or both (7, 39–44) for the screening or triage of HSIL and/or cervical cancer. In this validation trial, the performance of a DNA methylation assay based on the *EPB41L3* and *JAM3* genes was similar to that of our training set (26), and the assessment showed favorable results in identifying CIN2+, including ADC. These results agreed with those of previous reports (38, 40, 45). Although the sensitivity of the methylation assay was lower than that of hrHPV testing, both assays had similar NPVs. Moreover, the methylation assay was superior to hrHPV testing in terms of specificity and PPV. Additionally, the methylation assay had a similar sensitivity and NPV to those for HPV 16/18 testing but a significantly improved specificity and PPV. Even in patients with negative hrHPV results, methylation had good sensitivity and specificity compared with previous reports (32). These findings support the independent role of methylation assays in cervical cancer screening, as supported by our training set (26), validation set, and studies from other authors (29, 40, 46). However, the low positivity rates for methylation in CIN2/3 must be improved to improve the effectiveness of methylation for differentiating CIN2+. In summary, the methylation assay is a promising triage tool in hrHPV+ women, or even an independent tool for cervical cancer screening.

In this trial, for both CIN2/3 and SCC without residual lesions after biopsy or resection, the expressions of DNA methylation were absent. This indicates that methylation testing can be utilized as a robust biomarker for residual disease, offering potential benefits in terms of prognosis and patient outcomes. This finding is interesting in that it suggests that DNA methylation is highly disease specific. In the report of van Baars et al. (47), in women with multiple cervical biopsies, *CADM1/MAL* methylation was associated with lesion severity and was lesion specific, appearing to be representatives of the worst lesion, such as CIN3 or cervical cancer. Although the proportions of hrHPV positivity decreased significantly for CIN2+ without residual lesions compared with CIN2+ with residual lesions, hrHPV testing was not as good as the methylation assay for differentiating the two entities. In fact, in our study, for CIN2+ without residual lesions, the methylation levels were similar to those for benign diseases, but the proportions of patients with positive hrHPV testing results were still significantly higher for benign diseases. These findings suggest that DNA methylation could be a prognostic marker for CIN2/3 or SCC, as suggested by a prospective study (48). By incorporating methylation analysis into clinical practice, healthcare professionals can identify patients who are at a higher risk of residual disease. This information can guide treatment decisions, allowing for more targeted interventions and closer monitoring of those individuals. Early detection of residual disease through methylation testing can facilitate timely intervention, resulting in improved prognosis and enhanced patient management.

In the CIN2 group of our training set (47) and the current study, we found a lower rate of positive methylation (20.7% and 17.6%, respectively) than positive hrHPV results (72.4% and 76.5%, respectively). These differences led to decreased sensitivity of the methylation assay in the context of CIN2+. Host-cell DNA methylation patterns in cervical scrapings from women with CIN2 and CIN3 can be heterogeneous (49). Some authors have shown that methylation assays have higher NPVs but lower PPVs in CIN3+ than in CIN2+ (7). A DNA methylation panel of host and HPV gene (S5 classifier) classifiers showed high potential as a prognostic biomarker to identify progressive CIN2 (50). These findings support the use of methylation pattern assessments together with other diagnostic procedures, including immunostaining for specific markers, for in-depth analyses of CIN2 (51, 52).

In our training set (47) and the current study, we observed good diagnostic accuracies for methylation assays or hrHPV testing in cervical ADC. Unsurprisingly, methylation was best for identifying endocervical ADC, the most common type of ADC. According to the International Endocervical Adenocarcinoma Criteria and Classification (IECC) (53), ADCs of the in situ, endocervical, mucinous or villoglandular types are HPV-associated ADCs. ADC and SCC can have differential methylation patterns. Aberrantly high methylation of ZNF582 might even be a potential biomarker for determining ADC prognosis and chemoradiotherapy resistance (54). Therefore, a larger sample with multiple subtypes of ADC is needed to verify and confirm the differences between methylation assays and hrHPV testing for ADC diagnosis.

A number of studies have reported the favorable performance of DNA methylation assays for endometrial cancer tissues (55–57), ovarian cancer tissues (58–60), or both (61, 62). In our current study, we enrolled more patients with endometrial and ovarian tumors. Regarding ovarian tumors, only 12.2% of patients with EOC were positive for methylation; however, DNA methylation was detected in more than half (55.0%) of endometrial carcinomas. These differences reflect the limitations of cytological pathology in the diagnosis of endometrial or ovarian tumors. To assess methylation in uterine malignancies, an intrauterine tool might be able to obtain a larger number of methylation-positive cells than a cervical swab. There is very limited evidence of methylation in uterine or ovarian precancerous lesions. Overexpression of DNA methyltransferase 1 microRNA was mainly observed in endometrial carcinomas rather than in normal tissues or EINs (63). In the study by Marichereda et al. (64), methylation of the SFRP2 gene in the endometrial tissues of patients with hyperplastic processes was greater than 20-25%. However, gene ontology analysis has shown differential methylation at linked CpG sites between low-grade serous carcinomas and serous borderline tumors (65).

A strength of this study is its relatively large cohort with unselected gynecologic diseases. The detailed pathological results before and after major surgery (also before and after methylation assay) provided robust validation of *EPB41L3* and *JAM3* as tools for cervical and even endometrial lesion screening. However, there are several limitations of our study. First, we did not compare the methylation assay results to the cytology results. Since most patients with cervical lesions underwent cervical procedures before the last surgery (median time 40 days), the results of the cytology assay would likely be unaffected by surgery. DNA methylation analysis of HPV-positive self-obtained samples was not inferior to cytology triage in the detection of CIN2+ (66). The effectiveness of methylation assays versus cytology with or without hrHPV testing should be assessed in community-based populations in the future. Second, we did not follow up on the prognoses of the patients after surgery, limiting the interpretation of DNA methylation results in the carcinogenesis and progression of cervical cancer (67). Third, we excluded patients who were positive for HIV or pregnant women from the training and validation sets. This limitation could have hampered the extrapolation of the DNA methylation assay to a large cohort, as reported previously (68, 69).

In conclusion, in this validation trial, we discovered that methylation of *EPB41L3* plus *JAM3* had favorable diagnostic accuracy for CIN2+ and that it could be an independent screening method regardless of hrHPV status. The methylation assay was also sensitive to residual CIN2+, cervical and uterine adenocarcinomas.

## Data availability statement

The original contributions presented in the study are included in the article/**Supplementary Material**. Further inquiries can be directed to the corresponding author.

## Ethics statement

The institutional review board of Peking Union Medical College Hospital approved this study (No. JS-1954). The registration number is NCT03960879 (*clinicaltrials.gov*, registered on May 23, 2019).

## Author contributions 

LL and PL conceived of the original idea for the study, interpreted the results, performed the statistical analysis, edited the paper and were the overall guarantors. LK, LW, ZW, XX and LL obtained ethical approval, collected study samples, contributed to the preparation of the data set, interpreted the results and contributed to drafting the paper. LW, ZW and PL performed the DNA methylation analysis. YY and HW conducted the pathological evaluations. XX and MW contributed to the study design and interpretation of the results and commented on drafts of the paper. All of the authors have approved the final version of the manuscript.
